# Prospective study examining the probability of cerebral fat embolism based on magnetic resonance imaging

**DOI:** 10.1016/j.heliyon.2023.e14073

**Published:** 2023-02-26

**Authors:** Norihide Kanda, Takahito Miyake, Hideshi Okada, Yosuke Mizuno, Masahiro Ichihashi, Yoshinori Kakino, Tetsuya Fukuta, Yuichiro Kitagawa, Ryu Yasuda, Kodai Suzuki, Yukichi Tanahashi, Tomohiro Ando, Takahiko Asano, Takahiro Yoshida, Shozo Yoshida, Masayuki Matsuo, Shinji Ogura

**Affiliations:** a Department of Emergency and Disaster Medicine, Gifu University Graduate School of Medicine, 1-1 Yanagido, Gifu, 501-1194, Japan; b Department of Radiology, Gifu University Hospital, 1-1 Yanagido, Gifu, 501-1194, Japan; c Abuse Prevention Centre, Gifu University Graduate School of Medicine, 1-1 Yanagido, Gifu, 501-1194, Japan

**Keywords:** Cerebral fat embolism, Long-bone fracture, Pelvic fracture

## Abstract

**Purpose:**

Cerebral fat embolism (CFE) is a rare syndrome caused by the embolization of fat particles into the brain circulation. This prospective single-center observational study investigated the incidence of CFE in long-bone or pelvic fractures based on magnetic resonance imaging (MRI) findings. The purpose of this study was to investigate the incidence of CFE by MRI findings with or without symptoms suggestive of CFE.

**Methods:**

Eligible patients were consecutive, aged 15 years or older, with high-energy traumas, including pelvic or femur fractures. Excluded patients were those who died, could not undergo MRI resulting from medical conditions, or had insufficient mental capacity and no consultee to provide consent. The MRI was scheduled within 4 weeks of the injury, and the images were reviewed by one of the three neuroradiologists who were unaware of the patient's clinical information. Patient data regarding demographics, preceding trauma, injury severity score (ISS), presentation and examination timing of MRI, management including surgery, and outcome were collected.

**Results:**

Sixty-two patients were recruited, and three patients were excluded. All patients were injured by blunt trauma. The median patient age was 44 years. The median ISS was 13, and 53 patients needed surgical fixation. There were 22 patients with long-bone fractures, all of whom received external fixation or intramedullary nailing on admission day. MRI was performed after a median hospital day of 18 days. Using MRI imaging, three (5.0%) patients were diagnosed with CFE, and three patients were suspected of CFE.

**Conclusions:**

This is the first study to prospectively examine the probability of CFE based on MRI. Since fat embolism syndrome (FES) is confirmed in patients without clinical symptoms, CFE may be more common in patients with trauma than currently believed. Therefore, studies to determine the diagnostic criteria combined with symptoms, MRI, or other objective findings are required in the future.

## Highlights

• CFE is possible in trauma patients with slight mental disturbances, delirium, or even alert patients.
• CFE is more common in patients with trauma than currently believed.
• New CFE diagnostic criteria combined with symptoms and other objective findings including MRI are needed.

## 1 Introduction

Fat embolism syndrome (FES) is a rare syndrome caused by the embolization of fat particles into multiple organs, including the skin, lungs, and brain, which are the organs most affected by FES [1]. Usually, FES is a self-limiting disease; fulminant cases of FES may present with acute respiratory distress syndrome, cardiac dysfunction, and possibly death [2].

FES typically manifests as a petechial rash, neurologic disturbances, and progressive respiratory insufficiency. FES typically presents 24–72 h after an initial injury, which is most commonly a traumatic long-bone fracture. The diagnosis of FES is primarily based on clinical symptoms and imaging findings and requires a high index of suspicion [1]. Multiple factors associated with FES have been identified, including long-bone and pelvic fractures, orthopedic procedures such as intramedullary nailing, and total knee or hip arthroplasty [1,[3], [4], [5], [6]].

In most cases, FES is primarily diagnosed clinically. The classic clinical triad of symptoms usually does not occur simultaneously, and a petechial rash is present in less than half of the cases. The signs and symptoms are variable and nonspecific and can be mistaken for other diseases. Common and well-accepted diagnostic approaches for FES include the criteria proposed by Gurd Wilson [6] as well as by Lindeque [7]. However, these diagnostic tools have not been validated in prospective studies; consequently, there is no clear evidence of their sensitivity and specificity, limiting the practical applications of these criteria.

Some studies have reported the incidence of FES. Stein et al. investigated the incidence of FES from the National Hospital Discharge Survey and reported that 0.12% of patients with an isolated fracture of the femur, tibia, fibula, pelvis, ribs, humerus, radius, or ulna developed FES [8]. They also reported that 1.29% of patients with multiple fractures that included the femur (excluding neck) had FES.

Some prospective, consecutive studies of FES have been reported [4]; Fabian et al. reported in 92 patients that when hypoxemia follows long-bone or pelvic fracture, the rate of incidence for FES was 11% [9]. Chan et al. reported an 8.75% FES rate in consecutive trauma patients and a rate of 35% in patients with multiple injuries [10].

Among the reports on FES, there are minimal data on the estimated incidence of cerebral fat embolism (CFE) [1]. Armstrong et al. reported that the incidence of CFE was 0.04% in the trauma patients admitted to their hospital [11]. Kellogg et al. reported 54 cases of CFE and FES in 38 reports [12]. Using magnetic resonance imaging (MRI), they reported a 44.4% rate of incidence for CFE, following fracture injuries in the reviewed reports. The rate of CFE was reduced to 38.9% following early open reduction and fixation.

The diagnosis of FES or CFE is often clinical and nonspecific, and missed diagnosed cases are possible. Although CFE has sometimes been described as “a self-limiting disease”, it can be fatal and finding the true incidence of CFE is a major concern. The aim of this study was to investigate the incidence of CFE in long-bone or pelvic fractures based on MRI findings. To our knowledge, this is the first prospective study to examine the probability of CFE based on MRI.

## 2 Materials and methods

### 2.1 Study design and participants

This was a prospective, single-center observational study in Gifu University Hospital (Gifu-shi, Japan). The hospital is the only advanced critical care center in this region. The region includes catchment areas populated by approximately two million people. The attending emergency physicians were responsible for the trauma survey and treatment of patients in the emergency department (ED). The participants were enrolled between April 2016 and March 2020. Institutional and ethical approval (Gifu University Ethics Review Committee), including the method of consent and management of MRI results, were granted before recruitment.

**Fig. 1:**
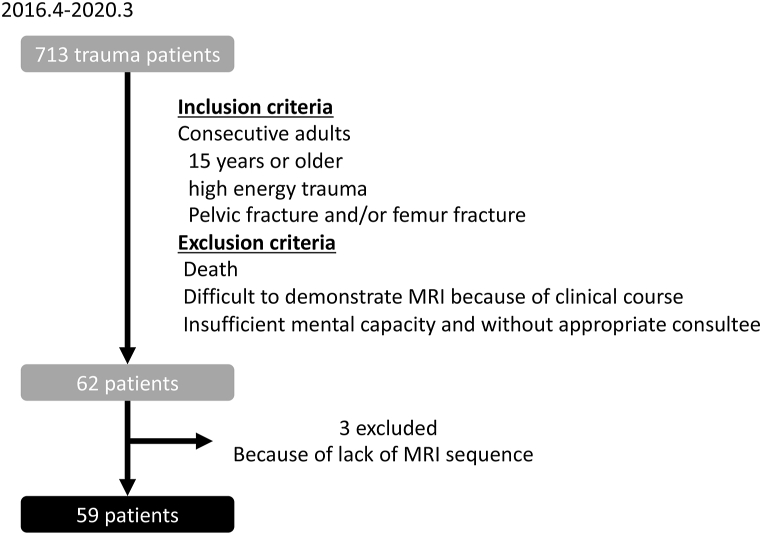
Study design and participants.

Eligible patients were 15 years or older consecutive patients with high-energy trauma, including pelvic or femur fractures, aged, admitted to the Advanced Critical Care Centre, Gifu University with or without neurological symptoms. They were able to provide fully informed written consent or, in the absence of the mental capacity an appropriate consultee provided written consent. Patients who died, or for whom it was difficult to obtain useful MRI results because of their clinical treatments (for example, electric devices attached to patients or patients not sufficiently stable for MRI), or those with insufficient mental capacity and without appropriate consultees were excluded (Fig. 1). The timing of each case's MRI were dependent on the clinical course: respiratory failure with respirator, disturbances of consciousness, and reasons for treatment schedule of other injuries.

### 2.2 Procedures

All patients were treated using the (advanced trauma life support) approach or specific therapies that are commonly used for each injury. All patients underwent a head CT scan on admission as part of the trauma survey. MRI was scheduled for 2–4 weeks after the injury and performed using a 3.0-T MRI system (Achieva Quasar Dual 3 T or Ingenia 3.0 T CX; Philips Medical Systems, Best, Netherlands) for 47 patients and a 1.5-T MRI system (Intera Achieva 1.5T Pulsar or Ingenia Prodiva 1.5 T CX; Philips Medical Systems, Best, Netherlands) for the remaining 13 patients. Susceptibility-weighted images (TR/TE, 23–34/39–54 msec; slice thickness, 1.2 mm; flip angle, 15°) were obtained in 42 patients. All MRI images, except susceptibility-weighted images, were obtained in the transverse plane at a section with a 5 mm thickness, a 1.5-mm intersection gap, and a field of view of 240 × 240 cm. T2*-weighted gradient-echo images (TR/TE, 506–639/16–23 msec) and T2 weighted spin-echo images (TR/TE, 3499–4842/88–100 msec) were obtained in 31 and 44 patients, respectively. Diffusion-weighted images (TR/TE, 2237–4000/67–88 msec) and fluid-attenuated inversion recovery (TR/TE, 6000–11,000/120 msec) were obtained for all patients. Diffusion-weighted images were acquired using a single-shot echo-planar imaging (EPI) sequence. The b values corresponding to the diffusion-sensitizing gradients were 0 and 1000 s/mm^2^.

Three MRI devices were used. All MRI images were reviewed by one of the three neuroradiologists (Y.T., T.A., and T. A.) who were blinded and had no access to clinical information. In this study, diagnostic cases were defined as the presence of a typical MRI imaging pattern of CFE known as the “starfield pattern.” Suspected cases were defined as lesions that suggested micro-emboli on MRI imaging and/or did not have the "starfield pattern."

Data were collected on patient demographics, history of preceding trauma, injury severity score (ISS), presentation and timing of MRI, care management, including surgery, and patient outcome. In addition, for patients who were diagnosed with FES, the results from the diagnostic criteria were added.

### 2.3 Outcome

The primary endpoint of the study was the incidence of CFE in high-risk patients examined by MRI, with or without clinical symptoms. High-risk patients were defined as trauma patients with femoral or pelvic fractures.

## 3 Results

### 3.1 Clinical outcomes

Between April 2016 and March 2020, 713 patients who were registered in the Japan Trauma Data Bank from Gifu University Hospital were admitted to the hospital, of whom 62 were recruited. Three patients were excluded due to a lack of MRI sequences. The median patient age was 44 (interquartile range [IQR]: 31–59) years, with 45 of 60 patients (76%) being men. All patients had blunt trauma injury: 28 in traffic accidents (motorcycle crashes, 12; vehicle crashes, 9; hit by a vehicle, 5; bicycle crashes, 2), 19 in a fall from a height, 7 in sports accidents, and 5 were injured by other means. 24 patients had pelvic ring fractures, 14 had acetabular fractures, 14 had femur fractures, 1 had pelvic ring and acetabular fractures, 4 had pelvic ring and femur fractures, 1 had acetabular and femur fractures and 1 had a sacral fracture (Table 1). There were no patients who were suggestive of a CFE by the primary head CT scan.

**Table 1:** Patient demographics, mechanism of injury, complicated injuries, fracture characteristics, timing, and operation details.

Demographic data of the patients	Median or number
Age	44 (31–87) years
Female/Male	14/45
Mechanism of injury	
Traffic accident	28
Fall from a height	19
Sports injury	7
Others	5
Complicated injuries	
Number of patients with complicated injuries	38
Head	7
Face	4
Chest	15
Abdomen	8
Spine	13
Fracture type	
Pelvic ring fracture	24
Acetabular fracture	14
Femur fracture	14
Pelvic ring + Acetabular fracture	1
Pelvic ring + Femur fracture	4
Acetabular fracture + Femur fracture	1
Sacral fracture	1
Timing of 1st operation (Total N = 53)	
Day 1	28 (EF: 15)
Day 2–3	4 (EF: 4)
Day 4–7	9 (EF: none)
Day 8–13	12 (EF: none)
Timing of surgery for femoral fractures	
Day 1–2: IMN/EF	14/8
Day 3–7: IMN/EF	5/1
Day 8–13: IMN/EF	3/0

IMN, intermedullary nailing; EF, external fixation.

The median ISS was 13 (IQR: 9–22). No fatalities occurred, and 53 patients needed surgical fixation for pelvic or femoral fractures. There were 22 patients with femoral fractures, and all of them underwent external fixation (EF) or intramedullary nailing (IMN) on the day of admission; among these, 14 had IMN. There were 22 patients who had an altered level of consciousness on admission, 7 with head trauma (abbreviated injury score: AIS ≧ 3), and 15 without head trauma among them. Of the 15 patients, there were 3 patients who were under sedation and intubation for hemorrhagic shock, and 4 patients who had mental disorders. We performed an thencephalogram on one comatose patient who was diagnosed with a CFE by MRI, and there was no obvious abnormal finding. There were no patients who were diagnosed with acute encephalopathy, focal neurological deficits, or seizures. The timing of surgery is summarized in Table 1.

**Fig. 2:**
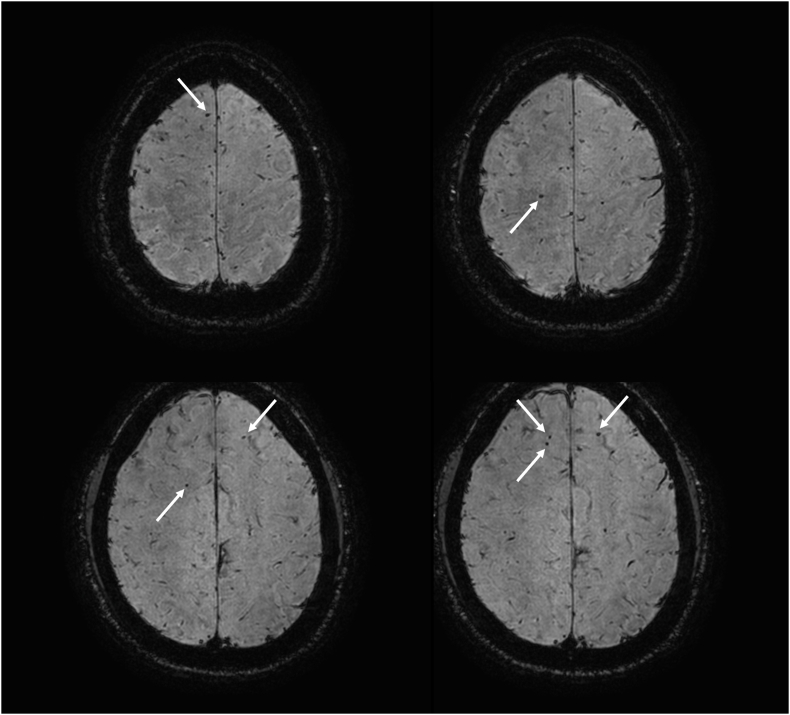
Suspected case of cerebral fat embolism (CFE) in a 38-year-old man who was injured due to a fall and diagnosed with right acetabular fracture and right kidney injury. The diagnostic criteria for fat embolism syndrome (FES) were not applicable, and there were no neurological problems. A brain magnetic resonance imaging (MRI) was performed on day 16. There were many low-intensity signs (white arrow), indicating small hemorrhages in the bilateral white matter of the frontal lobe on susceptibility-weighted imaging (SWI), which was assessed as diffuse axonal injury or CFE.

The median day at which an MRI was performed was 18 days (IQR: 11–21). Using MRI, three (5.0%) patients were diagnosed with CFE, and three patients were suspected of having CFE. The characteristics of these six cases are shown in Table 2. A magnetic resonance image of a patient suspected of having CFE is shown in Fig. 2. All of the population but two patients, who are described in case no.2 and no.3 indicated in Table 2, had no neurological symptoms. The patients who were diagnosed with suspected CFE did not receive specific treatment for CFE.

**Table 2:** Demographic data of diagnosed and suspected cases of cerebral fat embolism (CFE) by magnetic resonance imaging (MRI).

Case No.	Age	Sex	Mechanism of injury	Medical history	Pelvic/Femur fractures	Other injuries	ISS	Operation	MRI day	MRI Findings	Diagnostic criteria
Diagnosed cases by magnetic resonance imaging											
1	42	M	Hit by falling objects	Hyperlipidemia	Femur	Lumber fracture, Humeral fracture,	34	EF for femur (Day 0) ORIF for femur (Day 3)	26	Starfield pattern	Gurd's criteria: Major: Altered mentation, Hypoxia Minor: tachycardia Schonfeld's criteria: None Lindeque criteria: None
2	87	F	Hit by vehicle	Chronic heart failure	Femur (bilaterally) Pelvis	Traumatic SAH, Tibial fracture	18	EF for pelvis, ORIF for rt femur (Day 0) ORIF for lt femur (Day 5) ORIF for pelvis (Day 12)	24	Starfield pattern	Gurd's criteria: Major: Altered mentation Schonfeld's criteria: Hypoxia, mental confusion, hypoxia Lindeque criteria: Tachypnea
3	67	F	Hit by vehicle	Schizophrenia	Pelvis Tibia	None	5	None (ORIF for ankle fracture, Day 10)	2	Starfield pattern	Gurd's criteria: Major: Altered mentation, Hypoxia Major: tachycardia, fever, unexplained anemia Schonfeld's criteria: Hypoxia, fever, tachycardia Lindeque criteria: None
**Suspected cases by magnetic resonance imaging**											
4	66	M	Motor bicycle	None	Pelvis	Radial fracture, Tibial fracture	25	ORIF for pelvis (Day 12)	20	Petechial hemorrhage of white matter	Gurd's criteria: None Schonfeld's criteria: None Lindeque criteria: None
5	38	M	Fall from height	None	Pelvis	Rt kidney injury	14	ORIF for pelvis (Day 6)	16	Petechial hemorrhage of white matter	Gurd's criteria: None Schonfeld's criteria: None Lindeque criteria: None
6	28	M	Fall from height	Schizophrenia	Pelvis	Rt hemothorax and pneumothorax	41	ORIF for pelvis (Day 8)	19	Petechial hemorrhage of white matter	Gurd's criteria: Minor: tachycardia, Schonfeld's criteria: Tachycardia, mental confusion, tachypnea Lindeque criteria: None

ISS, injury severity score; EF, external fixation; ORIF, open reduction and fixation; SAH, subarachnoid hemorrhage; Rt, right.

## 4 Discussion

Although the exact mechanisms of FES are not known, two theories have been suggested: the “Mechanical Theory” and the “Biochemical Theory [4,[13], [14], [15]]”. The mechanical theory postulates that external mechanical forces such as traumatic injury or invasive surgery cause increased intramedullary pressure. This causes fat droplets to flow into the venous system. These fat emboli become lodged first in the pulmonary microvasculature, and then in the arterial circulation through right-to-left shunts such as in patent foramen ovale and arteriovenous shunts [14]. Eventually, they can cause multi-organ dysfunction. The biochemical theory proposes a biomechanical component in the pathophysiology of this syndrome. Biomechanical changes, including fat globules entering the pulmonary vasculature where they are broken down to free fatty acids, lead to a microvascular inflammatory response that results in acute lung injury [15]. At the brain level, fat droplets cause local ischemia and inflammation associated with the release of inflammatory mediators, leading to neurological signs and symptoms.

As an FES diagnosis is mainly based on clinical features, and most laboratory tests are nonspecific; imaging examination would facilitate the diagnosis of these syndromes. Chest radiology or chest computed tomography can be used to examine the lungs. Brain computed tomography or MRI is helpful in diagnosing CFE. According to Kuo et al., an MRI of the brain using T2-weighted, diffusion-weighted imaging (DWI), gadolinium contrast-enhanced images, and susceptibility-weighted imaging (SWI) has an improved the ability to make a diagnosis [16]. Consequently, when CFE is clinically suspected, especially at an early stage after the onset of neurological symptoms, rapid diagnosis by performing DWI and SWI is vital. Kuo et al. also presented five distinctive MRI patterns for CFE, which depended on the onset time. Furthermore, the most well-known pattern of CFE, the “starfield pattern,” is a nonspecific feature observed in many embolic events, and it is often reversible. In addition, it is possible for micro-emboli, which are small enough to pass through the pulmonary capillary bed, to cause CFE without right-to-left shunts [16]. Based on the literature, suspected cases in this study demonstrated the “petechial hemorrhage of white matter” pattern. Timings of the MRI from admission were relatively late, which could be a controversial point. Different studies on CFE imaging are sometimes conflicting, with different descriptions of timing, distribution, size, and reversibility of the brain lesions.

In this study, 5.1% (3/59) of the femur or pelvic fracture patients were diagnosed, and 5.1% (3/59) were suspected with CFE by MRI. As CFE is essentially an embolic shower of fat globules, the imaging findings could be confused with micro-infarction or micro-bleeding of cerebral arteries. The summary of FES incidence is described in Table 2. Among the three patients who were diagnosed with CFE by MRI, their consciousness was almost clear in the emergency room (ER), but an obvious disorder of consciousness appeared over time; subsequently, the typical starfield patterns on MRI were detected. We assert a reasonable probability of false-positive results during the clinical course of these patients. In contrast, two patients who were suspected of having CFE on MRI without any clinical symptoms described in the three major diagnostic criteria. Although these were not typical signs of CFE, there was a possibility of non-symptomatic CFE. Millroy et al. described that fat embolism is common at autopsy in trauma cases; an incidence rate of 75%–100% for FES was indicated in autopsy reports [15]. This may indicate a higher possibility of CFE in patients with trauma. While there have been few pathological analyses of CFE in autopsy cases [17], there are currently no reports on the incidence of CFE in general trauma patients. Our findings suggest that, for patients with slight mental disturbances, delirium, and even for alert patients, without completion of diagnostic criteria, there is a probability of CFE.

Generally, prevention of CFE/FES involves controlling or eliminating risk factors as soon as possible (within the first 24 h) after injury. Such strategies include immobilization, stabilization, external or internal fixation, and optimal treatment strategies for long-bone fractures [4]. In addition, it has been concluded that early fracture stabilization corresponds to a reduction in the mortality rate for patients with multiple injuries [18]. Through this study's discussions on the early total/appropriate care [19] or damage control orthopedics [[20], [21], [22]], we have performed surgical internal/external fixations of fractures as early as possible (patients were treated with EF for the first operation), and we made efforts to convert fixation into early internal fixation based on the general condition of the patients. Thus, in this study, 52% (28/54) of patients who required bone fixation were treated on the first day of hospital admission. All fixation procedures for pelvic or long-bone fractures were completed by day 13. How this treatment strategy influences the results is currently unknown, and future studies are required.

Our study has some limitations. First, an MRI was not performed simultaneously after the traumatic event or surgery for each patient. Second, this study had a small number of patients, as it was conducted at a single institution. Third, as many injuries have been indicated as risk factors for FES, we could not align the conditions of patients based on the type of injury, injured site, or ISS. Fourth, the timing of MRI execution (on average 18 days) and related patterns described in the manuscript are not necessarily those that are in the early stage and therefore useful for diagnostic purposes.

## 5 Conclusions

In this study, 5.0% (3/60) of the femur or pelvic fracture patients were diagnosed with CFE, and 5.0% (3/60) were suspected of having CFE by MRI, regardless of the presence of clinical symptoms. This may indicate that CFE is more common in patients with trauma than currently believed. As the diagnosis of FES/CFE is often clinical and should diagnosed earlier, physicians should consider that there is a possibility of CFE in trauma patients with slight mental disturbances, delirium, or even alert patients who might need an MRI. Currently, it is difficult to diagnose FES/CFE. Future studies to determine a diagnostic criteria combined with symptoms, MRI, or other objective findings are needed.

## Author contribution statement

Norihide Kanda, Takahito Miyake– Conceived and designed the experiments; Performed the experiments, Analyzed and interpreted the data and Wrote the paper.Hideshi Okada – Conceived and designed the experiments; Analyzed and interpreted the data and Wrote the paper.Yosuke Mizuno, Masahiro Ichihshi, Yoshinori Kakino, Tetsuya Fukuta, Yuichiro Kitagawa, Ryu Yasuda, Kodai Suzuki, Takahiro Yoshida, Shozo Yoshida– Performed the experiments.Yukichi Tanahashi, Tomohiro Ando, Takahiko Asano, Masayuki Matsuo, Shinji Ogura – Analyzed and interpreted the data.

## Funding statement

Takahito Miyake was supported by 10.13039/501100001691Japan Society for the Promotion of Science [19K18348].

## Data availability statement

Data will be made available on request.

## Declaration of competing interest

The authors declare that they have no known competing financial interests or personal relationships that could have appeared to influence the work reported in this paper.

## References

[1] Godoy D.A., Di Napoli M., Rabinstein A.A. (2018). Cerebral fat embolism: recognition, complications, and prognosis. Neurocritical Care.

[2] Kosova E., Bergmark B., Piazza G. (2015). Fat embolism syndrome. Circulation.

[3] Shaikh N. (2009). Emergency management of fat embolism syndrome. J. Emergencies, Trauma, Shock.

[4] Mellor A., Soni N. (2001). Fat embolism. Anaesthesia.

[5] Robert J.H., Hoffmeyer P., Broquet P.E., Cerutti P., Vasey H. (1993). Fat embolism syndrome. Orthop. Rev..

[6] Lee S.C., Yoon J.Y., Nam C.H., Kim T.K., Jung K.A., Lee D.W. (2012). Cerebral fat embolism syndrome after simultaneous bilateral total knee arthroplasty: a case series. J. Arthroplasty.

[7] Lindeque B.G., Schoeman H.S., Dommisse G.F., Boeyens M.C., Vlok A.L. (1987). Fat embolism and the fat embolism syndrome. A double-blind therapeutic study. J Bone Joint Surg Br.

[8] Stein P.D., Yaekoub A.Y., Matta F., Kleerekoper M. (2008). Fat embolism syndrome. Am. J. Med. Sci..

[9] Fabian T.C., Hoots A.V., Stanford D.S., Patterson C.R., Mangiante E.C. (1990). Fat embolism syndrome: prospective evaluation in 92 fracture patients. Crit. Care Med..

[10] Chan K.M., Tham K.T., Chiu H.S., Chow Y.N., Leung P.C. (1984). Post-traumatic fat embolism--its clinical and subclinical presentations. J. Trauma.

[11] Armstrong B.R.W., Devendra A., Pokale S., Subramani B., Rajesh Babu V., Ramesh P., Dheenadhayalan J., Rajasekaran S. (2022). Can the rate of mortality and neurological recovery be predicted from the time of onset of symptoms and MRI grade in patients with cerebral fat embolism? : a study of 34 patients. Bone Joint Lett. J.

[12] Kellogg R.G., Fontes R.B., Lopes D.K. (2013). Massive cerebral involvement in fat embolism syndrome and intracranial pressure management. J. Neurosurg..

[13] Hulman G. (1995). The pathogenesis of fat embolism. J. Pathol..

[14] Fukumoto L.E., Fukumoto K.D. (2018). Fat embolism syndrome. Nurs. Clin..

[15] Milroy C.M., Parai J.L. (2019). Fat embolism, fat embolism syndrome and the autopsy. Acad Forensic Pathol.

[16] Kuo K.H., Pan Y.J., Lai Y.J., Cheung W.K., Chang F.C., Jarosz J. (2014). Dynamic MR imaging patterns of cerebral fat embolism: a systematic review with illustrative cases. AJNR Am J Neuroradiol.

[17] Scully R.E. (1956). Fat embolism in Korean battle casualties; its incidence, clinical significance, and pathologic aspects. Am. J. Pathol..

[18] Bone L.B., McNamara K., Shine B., Border J. (1994). Mortality in multiple trauma patients with fractures. J. Trauma.

[19] Vallier H.A., Wang X., Moore T.A., Wilber J.H., Como J.J. (2013). Timing of orthopaedic surgery in multiple trauma patients: development of a protocol for early appropriate care. J. Orthop. Trauma.

[20] Guerado E., Bertrand M.L., Cano J.R., Cervan A.M., Galan A. (2019). Damage control orthopaedics: state of the art. World J. Orthoped..

[21] Scalea T.M., Boswell S.A., Scott J.D., Mitchell K.A., Kramer M.E., Pollak A.N. (2000). External fixation as a bridge to intramedullary nailing for patients with multiple injuries and with femur fractures: damage control orthopedics. J. Trauma.

[22] Blokhuis T.J., Pape H.C., Frolke J.P. (2017). Timing of definitive fixation of major long bone fractures: can fat embolism syndrome be prevented?. Injury.

